# Esophagus and Contralateral Lung-Sparing IMRT for Locally Advanced Lung Cancer in the Community Hospital Setting

**DOI:** 10.3389/fonc.2015.00127

**Published:** 2015-06-23

**Authors:** Johnny Kao, Jeffrey Pettit, Soombal Zahid, Kenneth D. Gold, Terry Palatt

**Affiliations:** ^1^Department of Radiation Oncology, Good Samaritan Hospital Medical Center, West Islip, NY, USA; ^2^Division of Hematology and Medical Oncology, Good Samaritan Hospital Medical Center, West Islip, NY, USA; ^3^Department of Surgery, Good Samaritan Hospital Medical Center, West Islip, NY, USA

**Keywords:** lung cancer, intensity-modulated radiation therapy, chemoradiation, toxicity, dose distribution

## Abstract

**Background:**

The optimal technique for performing lung IMRT remains poorly defined. We hypothesize that improved dose distributions associated with normal tissue-sparing IMRT can allow safe dose escalation resulting in decreased acute and late toxicity.

**Methods:**

We performed a retrospective analysis of 82 consecutive lung cancer patients treated with curative intent from 1/10 to 9/14. From 1/10 to 4/12, 44 patients were treated with the community standard of three-dimensional conformal radiotherapy or IMRT without specific esophagus or contralateral lung constraints (standard RT). From 5/12 to 9/14, 38 patients were treated with normal tissue-sparing IMRT with selective sparing of contralateral lung and esophagus. The study endpoints were dosimetry, toxicity, and overall survival.

**Results:**

Despite higher mean prescribed radiation doses in the normal tissue-sparing IMRT cohort (64.5 vs. 60.8 Gy, *p* = 0.04), patients treated with normal tissue-sparing IMRT had significantly lower lung V20, V10, V5, mean lung, esophageal V60, and mean esophagus doses compared to patients treated with standard RT (*p* ≤ 0.001). Patients in the normal tissue-sparing IMRT group had reduced acute grade ≥3 esophagitis (0 vs. 11%, *p* < 0.001), acute grade ≥2 weight loss (2 vs. 16%, *p* = 0.04), and late grade ≥2 pneumonitis (7 vs. 21%, *p* = 0.02). The 2-year overall survival was 52% with normal tissue-sparing IMRT arm compared to 28% for standard RT (*p* = 0.015).

**Conclusion:**

These data provide proof of principle that suboptimal radiation dose distributions are associated with significant acute and late lung and esophageal toxicity that may result in hospitalization or even premature mortality. Strict attention to contralateral lung and esophageal dose–volume constraints are feasible in the community hospital setting without sacrificing disease control.

## Introduction

Lung cancer remains the most common cause of cancer mortality in the United States with the majority of patients presenting with locally advanced or metastatic disease (1). Patients with locally advanced and oligometastatic lung cancer are commonly treated with chemoradiation with curative intent (2–4). Lung cancer treatment planning has significantly evolved over the past two decades (2, 5). RTOG 94-10 used three-dimensional conformal radiotherapy limiting the spinal cord to ≤48 Gy without specific lung or esophageal constraints (2). In the concurrent chemoradiation arm of RTOG 94-10, 23% of patients developed grade ≥3 esophagitis and 13% of patients developed grade ≥3 pulmonary toxicity (2). As the clinical significance of volume of lung receiving 20 Gy and mean lung dose became established, intensity-modulated radiation therapy emerged as a potential approach to reduce lung toxicity (6, 7). In RTOG 0617, either IMRT or three-dimensional conformal radiotherapy was allowed. RTOG 0617 strongly recommended a mean lung dose ≤20 Gy, volume of lung receiving 20 Gy ≤37%, and a mean esophagus dose of <34 Gy (5). Despite expanded use of normal tissue constraints, 20% of patients in RTOG 0617 developed grade ≥3 pulmonary toxicity. In RTOG 0617, 7% of patients receiving 60 Gy and concurrent chemotherapy developed grade ≥3 esophagitis, while 21% of patients receiving 74 Gy and concurrent chemotherapy developed grade ≥3 esophagitis (5). Maximum grade of esophagitis predicted survival on multivariate analysis (5). Taken together, these data suggest that IMRT technique for locally advanced lung cancer requires further refinement, particularly in the community oncology setting.

Although esophageal sparing was not highly prioritized in the past, there are emerging data linking weight loss and survival in patients with lung cancer treated with chemoradiation (8). A recent analysis linked volume of esophagus receiving 60 Gy with esophageal toxicity (9). The MD Anderson group has demonstrated the clinical significance of sparing the contralateral lung by limiting volumes of lung receiving 5 and 10 Gy (10). We investigated whether IMRT designed to limit lung V20, V10, V5, mean lung dose, and esophageal dose could reduce the risk of treatment toxicity compared to historical controls treated with usual care.

## Materials and Methods

### Patient selection

This retrospective study was approved by the Institutional Review Board. We reviewed 82 consecutive patients with stage II–III lung cancer, and patients with stage IV lung cancer with solitary oligometastases treated from January 2010 to September 2014. All patients had laboratory studies, computed tomography of the chest, and pathologic confirmation of malignancy. All patients had a complete metastatic workup with the vast majority of patients staged with whole body positron emission tomography and CT or MRI of the head. All patients were treated with potentially curative doses of ≥50 Gy for definitive radiation or ≥45 Gy in combination with surgery. Since the primary endpoints were dosimetry and toxicity rather than survival, no attempt was made to exclude patients based on histology or adjunctive treatment modalities (surgery, chemotherapy).

### Standard radiation technique

From January 2010 to April 2012, patients were treated by four highly experienced radiation oncologists using standard planning techniques. The most common technique was three-dimensional conformal radiotherapy alone (55%) although IMRT alone (24%) or in combination with 3D-CRT (21%) was also used. When IMRT was utilized, a median of five fields were used, with 33% of patients treated with seven to nine IMRT fields. During this period, the spinal cord was limited to a maximum dose of 50 Gy, the lung V20 was generally limited to ≤37%, and mean lung dose was ≤20 Gy. Patients were treated on a Varian 21C/D linear accelerator with CT-based planning with Pinnacle using tissue inhomogeneity corrections (Philips Medical Systems, Andover, MA, USA). Patients were immobilized in the supine position using a custom Alpha Cradle mold (Alpha Cradle Molds, Akron, OH, USA). Patients treated during this interval were classified as standard RT.

### Normal tissue-sparing IMRT technique

From May 2012 to September 2014, all patients were treated by a single high volume radiation oncologist (J.K.) with normal tissue-sparing IMRT. A minimum of 50 Gy was prescribed for small cell carcinoma and a minimum of 60 Gy was prescribed for non-small cell lung cancer. Further dose escalation was pursued only when dose constraints were not exceeded (Table 1). The most common technique used was IMRT alone (75%), usually a four-field technique. Patients with very bulky disease were treated with combined 3D-CRT (generally AP/PA or shallow obliques) in combination with IMRT (20%). Two patients (5%) with bilateral lung and/or hilar involvement, but no mediastinal involvement on PET, were treated with bilateral AP–PA fields as the optimal approach to lung and esophageal sparing.

**Table 1:** **Normal organ constraints used for normal tissue-sparing IMRT lung plans after May 2012**.

Lungs	Esophagus	Spinal cord	Heart	Brachial plexus
Dmean <20 Gy	Mean <34 Gy	Dmax <45 Gy	D60 <33%	Dmax <66 Gy
V20 <37%	Dmax <100% Rx (when feasible)	Spinal canal + 5 mm <50 Gy	D45 <66%	
V10 <50%			D40 <100%	
V5 <65%				

A median of 4 IMRT fields were used with 98% of patients treated with ≤5 fields. From May 2012 through January 2014, patients were treated on a Varian 21C/D with CT-based planning with PET/CT fusion. The gross tumor volume was defined as the primary tumor or any regional lymph nodes on CT (>1 cm on short axis) or PET. Fusion of inhalation, expiration, and free breathing CT scans were used to assess respiratory motion and to create an ITV. After February 2014, patients were treated on the Varian TrueBeam with 4-D CT simulation using Eclipse planning. ITV to CTV margins were 0.5–1.0 cm. CTV to PTV margins of 0.5–1 cm were used to create a PTV. In general, smaller margins within the range were used for bulky disease or tumor adherent to bone. When there was PET-positive disease involving the esophagus or vertebral bodies, the expanded PTV volumes were trimmed off critical structures to meet esophagus and spinal cord constraints.

A typical patient in the normal tissue-sparing IMRT group with a primary lung tumor with ipsilateral hilar, paratracheal, and/or aortopulmonary lymph nodes was usually treated to 64–70 Gy. When a final boost was utilized, the margins from GTV to boost PTV were limited to 1–1.2 cm in the superior inferior direction, 0.8–1.0 cm in the anterior posterior direction, and 0.5–0.8 cm in the medial lateral direction. Simultaneous integrated boost planning was performed to deliver radiation dose above 60 Gy to the bulky primary tumor and lymph nodes while administering a more conservative dose of 59.4–63 Gy to small volume CT or PET positive mediastinal lymph nodes measuring <2 cm that were in close proximity to the esophagus. For patients with contralateral mediastinal or hilar adenopathy, dose escalation beyond 60 Gy was usually not technically feasible. Although elective nodal irradiation was not performed, all PET positive lymph nodes and lymph nodes measuring >1 cm on short axis on CT were targeted for treatment.

### Follow-up

Patients were assessed weekly during radiation for toxicity, and weight was recorded. Following treatment, patients were reassessed at 1 month. Clinical follow-up, CT, and/or PET were performed at 3- to 4-month intervals for 2 years and at 4- to 6-month intervals thereafter. Patients were censored at last follow-up or death. Date of death was confirmed using the social security death index. Hospitalization was confirmed by reviewing the electronic medical record (EPIC).

### Toxicity scoring and statistical methods

Treatment-related toxicity was scored using the Common Terminology Criteria for Adverse Events version 4.0. Statistical analyses were performed on the entire cohort and for the predetermined subset of patients with stage II–III non-small cell lung cancer who were treated with definitive chemoradiation. Hospitalizations following treatment were documented in the EPIC electronic medical record. Differences in toxicity rates were assessed using a two-sided chi-square test with *p* values of <0.05 considered statistically significant. Local recurrence was defined as radiographic progression within the radiation volume that was not attributed to radiation fibrosis or pneumonitis. Actuarial locoregional control and overall survival were calculated using the Kaplan–Meier method from the initiation of radiation therapy.

## Results

### Patients and tumor characteristics

Table 2 summarizes the patient and tumor characteristics for the 38 patients treated with standard technique between 1/10 and 4/12 and the 44 patients treated with normal tissue-sparing IMRT between 5/12 and 9/14. Median follow-up time was 14 months (range 1.4–35.3 months). Both groups were well matched in terms of age, gender, histology, smoking history, race, clinical stage, performance status, pre-treatment weight loss, tumor location, and GTV volume. The median age for all patients was 69 with 53% male, 79% non-small small cell, 83% white, 70% ECOG 0-1, and 73% stage III. The mean GTV volume for patients receiving standard RT was 176.8 (range 13–615 cc) vs. 155.6 (range 4–803 cc) for IMRT patients (*p* = 0.52).

**Table 2:** **Patient characteristics for patients treated with standard RT or esophagus and contralateral lung-sparing IMRT**.

	Normal tissue-sparing IMRT (*n* **=** 44)	Standard (*n* **=** 38)	*p*
**Age (years)**			
Median	72	67	0.63
Range	30–87	48–83	
**Gender**			
Male	25 (56%)	17 (45%)	0.28
Female	19 (44%)	21 (55%)	
**Histology**			
Adenocarcinoma	18 (41%)	14 (37%)	0.29
Squamous cell carcinoma	15 (34%)	12 (32%)	
Small cell carcinoma	7 (16%)	10 (26%)	
Non-small cell lung cancer, NOS	4 (9%)	2 (5%)	
**Smoking (pack years)**			
Never	3 (7%)	1 (3%)	0.38
1–20	5 (11%)	13 (34%)	
21–40	14 (37%)	5 (13%)	
>40	22 (50%)	18 (47%)	
**Race**			
White	39 (89%)	29 (76%)	0.15
Non-white	5 (11%)	9 (24%)	
**Clinical stage**			
II	7 (16%)	4 (11%)	0.64
IIIA	14 (32%)	19 (50%)	
IIIB	16 (36%)	11 (29%)	
IV	7 (16%)	4 (11%)	
**Recurrent**			
No	37 (84%)	34 (89%)	0.48
Yes	7 (16%)	4 (11%)	
**ECOG performance status**			
0–1	27 (61%)	30 (79%)	0.12
2	12 (27%)	5 (13%)	
3	5 (11%)	3 (8%)	
**Weight loss**			
<10%	39 (89%)	34 (89%)	0.70
>10%	5 (11%)	4 (11%)	
**Location**			
Right upper	16 (36%)	10 (26%)	0.10 (upper vs. lower)
Right middle	5 (11%)	3 (8%)	
Right lower	9 (20%)	3 (8%)	
Left upper	9 (20%)	16 (42%)	
Left lower	4 (9%)	5 (13%)	
Lymph node only	1 (2%)	1 (3%)	

### Treatment characteristics

Table 3 summarizes treatment parameters. Most patients were treated with concurrent chemotherapy (87%) and without surgery (93%). Patients in the normal tissue-sparing IMRT group received a higher mean radiation (64.5 Gy ± SD 5.0 vs. 60.8 Gy ± 6.2, *p* = 0.04) and were significantly more likely to receive IMRT only compared to the standard group (75 vs. 24%, *p* < 0.001).

**Table 3:** **Treatment characteristics for patients treated with standard RT or esophagus and contralateral lung-sparing IMRT**.

	Normal tissue-sparing IMRT (*n* **=** 44)	Standard (*n* **=** 38)	*p*
**Radiation dose**			
Median	66	63	0.04
<59.4 Gy	6 (14%)	9 (24%)	
59.4–63 Gy	11 (25%)	16 (32%)	
63.1–66 Gy	11 (25%)	5 (13%)	
>66.1 Gy	16 (36%)	8 (21%)	
**Technique**			
IMRT only	33 (75%)	9 (24%)	<0.001
Combined 3D-CRT + IMRT	9 (20%)	8 (21%)	
3D-CRT only	2 (5%)	21 (55%)	
**Number of IMRT fields**			
Median	4	5	0.001
Range	3–7	4–9	
**Treatment time**			
Median	49	53	0.31
Range	37–68	29–180	
**Chemotherapy**			
Yes	36 (82%)	35 (92%)	0.07
No	8 (18%)	3 (8%)	
**Surgery**			
Yes	4 (9%)	2 (5%)	0.51
No	40 (91%)	36 (95%)	
**Lung volume receiving 20 Gy**	23.3% (SD ± 7.2)	32.2% (SD ± 11.6)	<0.001
Range	7–38%	10–58%	
**Lung volume receiving 10 Gy**	33.5% (SD ± 10.0)	45.7% (SD ± 15.4)	<0.001
Range	14–52%	15–79%	
**Lung volume receiving 5 Gy**	44.5% (SD ± 13.4)	61.2% (SD ± 18.9)	<0.001
Range	16–80%	19–99%	
**Mean lung dose**	14.0 Gy (SD ± 5.5 Gy)	17.6 Gy (SD ± 5.6 Gy)	0.005
Range	5.8–39.9 Gy	5.9–26.2 Gy	
**Maximum esophagus dose**	56.5 (SD ± 13.6 Gy)	61.1 (SD ± 14.0 Gy)	0.07
Range	6–68.3 Gy	45.8–70.0 Gy	
**Mean esophagus dose**	20.8 Gy (SD ± 10.9 Gy)	34.0 Gy (SD ± 13.7 Gy)	<0.001
Range	0.9–45.3 Gy	6.8–60.1 Gy	
**Esophageal volume receiving 60 Gy**	3.5 (SD ± 5.8 Gy)	14.5 (SD ± 16.4 Gy)	0.001
Range	0–19.0 Gy	0–58.0 Gy	
**Mean heart dose**	15.2 Gy (SD ± 10.4 Gy)	18.6 Gy (SD ± 9.8 Gy)	0.14
Range	0.7–39.1 Gy	0.7–34.3 Gy	
**Maximum spinal cord dose**	36.2 (SD ± 11.6 Gy)	42.1 (SD ± 9.3 Gy)	0.013
Range	2.1–46.5 Gy	4.9–49.5 Gy	

**Figure 1:**
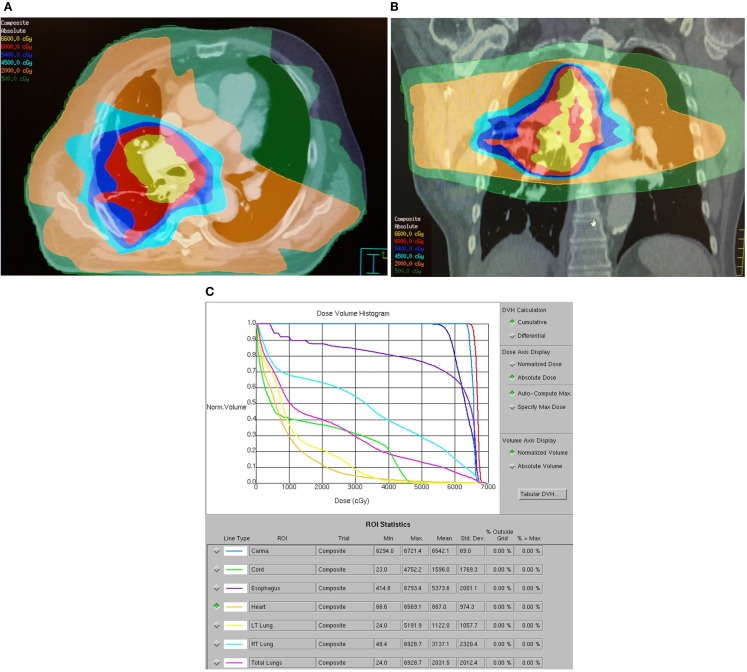
**Dose distribution for a representative patient with a 2.5 cm T3N0M0 stage IIB right lower lobe adenocarcinoma treated with standard RT technique**. The patient was treated with carboplatin, paclitaxel, and thoracic RT to 63 Gy via six-field IMRT followed by an eight-field IMRT boost. The patient developed acute grade 2 dysphagia and grade 1 dyspnea and died 8 months after treatment from acute myocardial infarction without evidence of progression. **(A)** Axial dose distribution demonstrates high target volume conformality but inclusion of a significant volume of esophagus within the high dose–volume. A significant volume of lung received at least 5 Gy. **(B)** Coronal dose distribution. **(C)** Dose–volume histogram demonstrates high-esophageal V60 and high lung V5, V10, V20, and mean lung doses.

**Figure 2:**
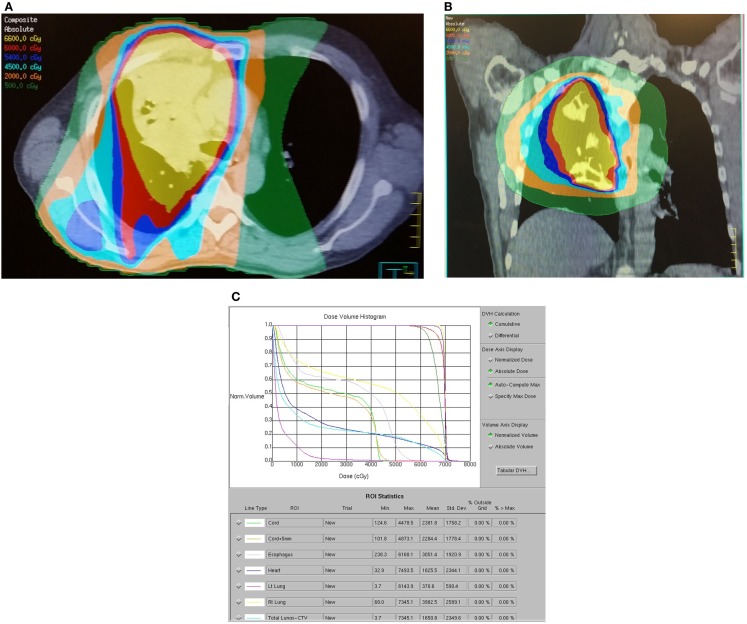
**Dose distribution for a representative patient with a 6.6 cm T4N2M0 stage IIIB right upper lobe adenocarcinoma treated with contralateral lung and esophageal sparing IMRT technique**. The patient was treated with carboplatin, paclitaxel, and thoracic RT to 73 Gy via four-field IMRT. The patient developed acute grade 2 dysphagia, grade 1 dermatitis, and right upper arm deep vein thrombosis. The patient remains alive and free of progression or late toxicity at 21 months. **(A)** Axial dose distribution demonstrates relatively poor conformality but excellent sparing of the esophagus and contralateral lung. **(B)** Coronal isodose distribution. **(C)** Dose–volume histogram demonstrates excellent coverage of the dominant mass (PTV70) and targeted lymph nodes (PTV63) with selective sparing of esophagus and lung.

The normal tissue-sparing IMRT cohort had significantly lower lung V20, V10, V5, mean lung, maximum esophagus, and mean esophagus doses (*p* ≤ 0.001). Mean lung V20 was 23.3 Gy ± SD 7.2 in the normal tissue-sparing IMRT group vs. 32.2 Gy ± SD 11.6 for standard RT. Mean esophagus dose was 20.8 Gy ± SD 10.9 with normal tissue-sparing IMRT vs. 34.0 Gy ± SD 13.7 for standard RT (*p* = 0.001). Representative dose distributions for standard RT and normal tissue-sparing IMRT are shown in Figures 1 and 2.

### Acute and late toxicity

The reduction in esophageal doses with normal tissue-sparing IMRT translated to significantly reduced incidence of acute grade ≥2 esophagitis (71 vs. 31%, *p* < 0.001). The incidence of acute grade ≥3 esophagitis was 0% with normal tissue-sparing IMRT compared to 11% with standard RT (*p* < 0.001). Average weight loss was 10.8 pounds ± SD 10.0 for standard RT vs. 4.8 pounds ± SD 5.6 for normal tissue-sparing IMRT (*p* = 0.003). Normal tissue-sparing IMRT reduced the risk of grade ≥2 weight loss (16 vs. 2%, *p* = 0.04). There were no significant differences in acute fatigue, nausea, lung, heart, or skin toxicity between treatment groups, and grade 3 toxicities were uncommon (<5%).

There was a significant reduction in grade ≥2 radiation pneumonitis with normal tissue-sparing IMRT (21 vs. 7%, *p* = 0.02). Three patients in the standard RT group-developed grade 5 pneumonitis had lung V20 values of 41, 48, and 58% and lung V5 values of 90, 99, and 88%, respectively. There was also one case of late grade 3 esophagitis and two cases of grade 3 pneumonitis with standard RT vs. none for normal tissue-sparing IMRT. The incidence of late grade ≥3 pulmonary toxicity was 0% with normal tissue-sparing IMRT compared to 14% with standard RT (*p* = 0.02). The incidence of hospitalization for dehydration and/or pulmonary symptoms within 6 months of treatment was 11% for normal tissue-sparing IMRT patients vs. 37% for standard RT (*p* = 0.008).

### Survival

**Figure 3:**
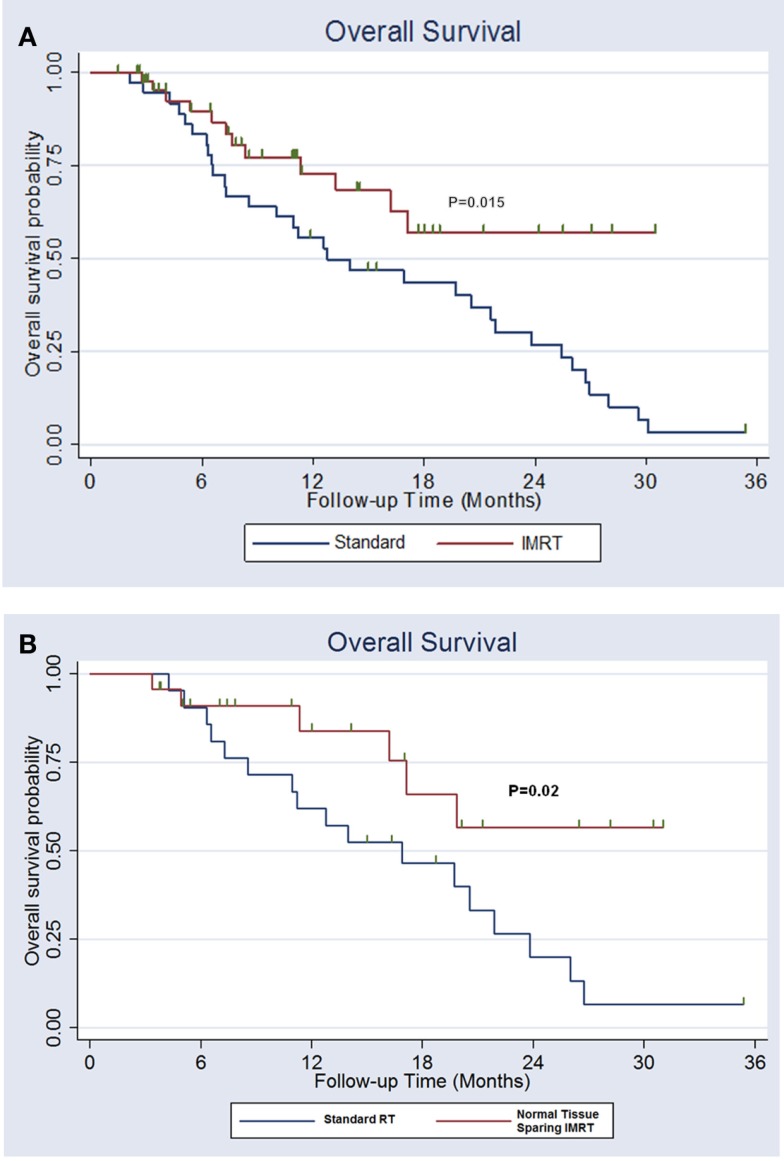
**(A)** Overall survival for lung cancer patients treated with esophagus and normal lung-sparing IMRT vs. standard technique. **(B)** Overall survival for patients with stage II–IIIB non-small cell lung cancer receiving definitive chemoradiation treated with esophagus and normal lung-sparing IMRT vs. standard technique.

The 2-year overall survival was 52% with normal tissue-sparing IMRT vs. 28% for standard RT (*p* = 0.015). The median survival for normal tissue IMRT was >31 months vs. 13 months for standard RT (Figure 3A). The 2-year locoregional control was 70% with normal tissue-sparing IMRT vs. 42% for standard RT (*p* = 0.12).

### Patients with stage II–III non-small cell lung cancer treated with definitive chemoradiation

A total of 43 patients had stage II–IIIB non-small cell lung cancer that was treated with definitive chemoradiation. The patients treated with normal tissue-sparing IMRT were more likely to receive IMRT, were treated with fewer IMRT fields, and had significant reductions in lung V20, V10, V5, mean lung dose, maximum esophagus dose, mean esophagus dose, and maximum spinal cord doses. Patients treated with normal tissue-sparing IMRT had reduced rates of acute ≥2 esophagitis (81 vs. 35%, *p* = 0.001), lower median weight loss (12.8 vs. 5.4 pounds, *p* = 0.01) and decreased late ≥2 radiation pneumonitis (19 vs. 0%, *p* = 0.04). There was a trend toward decreased hospitalization for dehydration and/or pulmonary symptoms within 6 months of treatment with normal tissue-sparing IMRT (33 vs. 12%, *p* = 0.12). The 2-year overall survival was 57% with normal tissue-sparing IMRT vs. 20% for standard RT (*p* = 0.02). The median survival for normal tissue IMRT was >31 vs. 16 months for standard RT (Figure 3B).

## Discussion

IMRT has been previously shown to reduce radiation dose to normal lung translating to reduced pulmonary toxicity for patients with locally advanced lung cancer (11). Although IMRT is increasingly used in locally advanced lung cancer, a clinical advantage over three-dimensional conformal radiotherapy remains unclear in population-based studies (12). The recently published RTOG 0617 trial allowed for IMRT for locally advanced lung cancer but a benefit for IMRT was not observed (5). As a result, some payers are reluctant to endorse IMRT for lung cancer (13). Skeptics have started to question the continuing focus on improving dose distributions in radiation oncology research (14). In terms of process improvements, expanded use of dose–volume constraints to improve outcome for patients requiring highly complex radiation treatment plans for locally advanced lung cancer could be considered analogous to use of checklists in other domains of medicine (15).

In the absence of randomized trials comparing normal tissue IMRT vs. standard RT, our study represents a natural experiment where lung cancer treatment technique abruptly transitioned from standard RT performed by four board-certified radiation oncologists to a single high volume radiation oncologist who implemented lung and esophagus-sparing IMRT (16). This study suggests that improved dose distributions resulting from adopting a policy of normal tissue-sparing IMRT favorably impacts outcome in lung cancer patients, including locally advanced non-small cell lung cancer compared to usual care by reducing high-grade lung and esophageal toxicity. The observed improvement in dosimetric endpoints translated into a clinically significant reduction in hospitalization for dehydration and pulmonary complications without sacrificing disease control. Patients treated with normal tissue-sparing IMRT had improved survival compared to patients treated with standard RT. Importantly, normal tissue-sparing IMRT could be safely performed with resources available within a community hospital cancer center.

The target volumes, organs at risk, and planning techniques for locally advanced lung cancers are diverse depending on location and size of the primary tumor and extent of lymph node involvement. Although there is not yet universal agreement on the clinical relevance of lung V10 and V5, the negative impact of bilateral low-dose (<15 Gy) lung irradiation has been documented in diverse conditions including mesothelioma, total lung irradiation for Wilm’s tumor, and total body irradiation for hematologic malignancies (17–19). The heterogeneity of IMRT planning approaches is reflected by published IMRT lung plans using between four and nine fields (20, 21). Typical IMRT plans utilize five or more coplanar beams that can result in up to 98% of the lung receiving 5 Gy for patients with large PTV volumes (7). Using five beams has been associated with reduced lung V5 compared to plans using seven or nine beams (22). Recently, five- to six-field IMRT plan was associated with a higher rate of grade ≥3 radiation esophagitis than three-dimensional conformal radiotherapy (23).

Since radiation pneumonitis is a potentially fatal complication, limiting contralateral lung exposure makes intuitive sense but is technically challenging (6). It is generally our preference to sacrifice conformality for contralateral lung avoidance by using four- to five-beam IMRT for the majority of patients. In cases where extent of disease could not be adequately covered by four- to five-IMRT fields, our preference has been to utilize limited field 3D-CRT (AP/PA or shallow obliques) for at least part of the treatment (24, 25).

Esophageal sparing is controversial because mediastinal lymph nodes, especially subcarinal, posterior paratracheal, and paraesophageal lymph nodes, often overlap with the esophagus. In this study, esophageal sparing was accomplished by simply avoiding the esophagus when there was no subcarinal or paraesophageal adenopathy on PET/CT. When subcarinal or paraesophageal lymph nodes were involved, these regions were not treated above 60 Gy by constraining esophageal V60 below prescription dose.

A central conundrum in lung cancer radiotherapy is that 60 Gy with concurrent chemotherapy is logically inadequate for bulky disease but efforts to further escalate treatment intensity using standard radiation techniques have been unsuccessful. RTOG 0617 implies that dose escalation above 60 Gy with concurrent chemotherapy is counterproductive due to increased toxicity (26). A prior CALGB trial using 66 Gy with concurrent chemotherapy ± induction chemotherapy resulted in poor outcomes in the cooperative group setting with survival (27). The notion that radiation dose intensification beyond 60 Gy in 6 weeks, a regimen established by RTOG 73-01, cannot be safely performed the era of IMRT, IGRT, and four-dimensional computed tomography simulation is counterintuitive (11, 28). Locoregional failure occurred in 31% of patients receiving 60 Gy and concurrent chemotherapy in RTOG 0617 (26). A potential solution explored in this study is administering the standard RTOG 0617 dose of 60 Gy to all areas of suspected involvement but selectively boosting bulky primary tumor and lymph nodes to 64–70 Gy. In this study, 61% of patients in the normal tissue-sparing IMRT cohort were treated to 64–70 Gy suggesting that selective dose escalation above 60 Gy can safely be performed in appropriately selected patients when using strict dose–volume constraints. Stereotactic radiotherapy boost represents a similar concept but remains experimental (29).

The authors acknowledge significant weaknesses inherent in a retrospective single-institution study based in a 437-bed community teaching hospital. The sample size was small and there was significant heterogeneity of the patient population in terms of stage, histology, treatment modality, and treatment technique. When stratifying results by stage (II–III vs. IV), histology (non-small cell vs. small cell), and treatment modality (chemoradiation vs. radiation alone), significant reductions in lung and esophageal dosimetry, esophagitis, weight loss, and radiation pneumonitis remained statistically significant. Small cell lung cancers were included because they were treated with radiation doses and volumes similar to non-small cell lung cancers. The survival in the standard RT arm was similar to results published in CALGB 39801, which is worse than expected for a modern cohort (27). Additionally, this study did not specifically evaluate whether the observed benefit was due to IMRT, four-dimensional CT simulation, PET/CT fusion, higher radiation dose, or physician experience but instead represented two cohorts separated in time and treated by different physicians with different normal tissue constraints. Despite these limitations, this study highlights the potential importance of technique in lung cancer radiotherapy in a real-world setting.

In conclusion, these hypothesis-generating data suggest that suboptimal radiation dose distributions associated with standard technique may result in significant acute and late lung and esophageal toxicity that result in hospitalization or even premature mortality. We suggest a relatively simple four-field IMRT technique with strict attention to contralateral lung and esophageal dose–­volume constraints for further validation as a potential class solution for many locally advanced lung cancers.

## Conflict of Interest Statement

The authors declare that the research was conducted in the absence of any commercial or financial relationships that could be construed as a potential conflict of interest.
